# NKG2D/CD28 chimeric receptor boosts cytotoxicity and durability of CAR-T cells for solid and hematological tumors

**DOI:** 10.1186/s40164-025-00646-3

**Published:** 2025-04-03

**Authors:** Xia Teng, Shance Li, Chaoting Zhang, Huirong Ding, Zhihua Tian, Yuge Zhu, Ting Liu, Guanyu Zhang, Kang Sun, Huimin Xie, Jiaxin Tu, Zheming Lu

**Affiliations:** 1 Key Laboratory of Carcinogenesis and Translational Research (Ministry of Education/Beijing), Laboratory of Biochemistry and Molecular Biology, Peking University Cancer Hospital & Institute, Beijing, 100142 China; 2 Key Laboratory of Carcinogenesis and Translational Research (Ministry of Education/Beijing), Central Laboratory, Peking University Cancer Hospital & Institute, Beijing, 100142 China; 3 Key Laboratory of Carcinogenesis and Translational Research (Ministry of Education), Department of Thoracic Surgery II, Peking University Cancer Hospital & Institute, Beijing, 100142 China

**Keywords:** CAR-T cell therapy, NKG2D/CD28 chimeric costimulatory receptor, CAR-T cell persistence, Low-density antigen expression

## Abstract

**Background:**

CAR-T cell therapy faces challenges in solid tumor treatment and hematologic malignancy relapse, among which the limited persistence of CAR-T cells and target antigen downregulation are prominent factors. Therefore, we engineered an NKG2D/CD28 chimeric co-stimulatory receptor (CCR), leveraging its broad ligand expression on tumors to enhance the antitumor activity of MSLN CAR and CD19 CAR-T cells.

**Methods:**

We generated MSLN CAR-T and CD19 CAR-T cells co-expressing the NKG2D/CD28 CCR and assessed their antitumor efficacy in vitro and in vivo. CAR-T cell activation, differentiation, and exhaustion were analyzed over time following tumor antigen stimulation. Furthermore, a chronic antigen stimulation model was established using tumor cells with low antigen density to simulate the sustained antigenic pressure encountered in vivo treatment conditions.

**Results:**

Our study shows that NKG2D/CD28&CAR-T cells exhibit enhanced cytotoxicity against tumor cells, especially those with low antigen density, both in vitro and in vivo. Compared to conventional second-generation MSLN CAR or CD19 CAR-T cells, these dual-targeted NKG2D/CD28&CAR-T cells demonstrate superior sensitivity in recognizing and lysing low-density antigen-expressing lung cancer and leukemia cells, and they are capable of eradicating tumors with low-density antigen expression in vivo. Furthermore, the complementary co-stimulation provided by the 4-1BB and CD28 intracellular domains in the CAR and NKG2D/CD28 promotes cytokine secretion, reduces CAR-T cell exhaustion, and enhances the in vivo persistence of CAR-T cells, significantly improving their antitumor efficacy.

**Conclusion:**

The combination of CAR and NKG2D/CD28 offers a potent strategy to enhance the cytotoxicity and durability of CAR-T cells. This approach is promising for improving therapeutic outcomes in solid and hematological tumors and preventing recurrence in tumors with low target antigen density.

**Supplementary Information:**

The online version contains supplementary material available at 10.1186/s40164-025-00646-3.

## Background

Chimeric antigen receptor (CAR) T cell therapy has revolutionized cancer treatment, demonstrating remarkable efficacy particularly in hematological malignancies such as leukemia and lymphoma [1]. The therapy involves genetically engineering a patient’s T cells to express a receptor that targets a specific antigen on cancer cells, enabling the immune system to recognize and destroy these cells with precision. Despite the promising advancements, CAR-T therapy confronts challenges that limit its broader application, notably the issue of antigen density expression and the durability of CAR-T cell activity [2–4].

The efficacy of CAR-T cells is dependent on the expression density of the target antigen, with a minimum threshold required to sustain effective T cell activity [5]. When antigen expression falls below this threshold, CAR-T cells may lose their functionality, a challenge that is particularly pronounced in tumors with heterogeneous antigen expression. To address this issue, strategies such as multi-specific targeting, including the use of bispecific or tandem CARs, have been proposed to tackle tumor heterogeneity and prevent antigen escape [6, 7].

The persistence of CAR-T cells post-treatment is crucial for achieving long-term therapeutic effects [8]. Researchers are currently concentrating on enhancing the durability and efficacy of CAR-T cell therapy. One strategy involves genetically modifying CAR-T cells by equipping them with chimeric co-stimulatory receptors (CCRs) to provide a sustained activation signal, thereby enhancing expansion and survival rates post-infusion [9–12]. For instance, Katsarou et al. demonstrated that combining a CAR with a CD38-directed CCR can improve T cell sensitivity to low antigen density and promote persistence [9].

In this context, our research has focused on developing a novel CCR, the NKG2D/CD28 receptor, designed to enhance the anti-tumor efficacy of CAR-T cells by providing a more potent and sustained activation signal. The NKG2D component of the receptor can recognize a broad range of tumor cells that express NKG2D ligands (NKG2DLs), which are often upregulated under stress conditions, such as in senescent cells or tumor cells [13]. Our previous research has highlighted the potential of NKG2D ligands as attractive tumor targets [14]. Although their high expression on tumors makes them ideal targets for CAR-T therapy, the presence of these ligands on certain normal or damaged cells raises concerns regarding potential off-tumor toxicity associated with NKG2D CAR-T therapy [15, 16]. Nevertheless, their broad high expression on tumors and ability to effectively activate the NKG2D receptor position them as promising candidate for a co-stimulatory receptor target. Meanwhile, the CD28 component delivers the essential second signal required for T cell activation, proliferation, and survival [17]. This co-stimulatory signal is vital for preventing CAR-T cell exhaustion and enhancing their capacity to recognize and destroy cancer cells [18]. Our previous work with CAR-T cells incorporating a PD-1/CD28 receptor has shown the advantages of this approach [19]. By harnessing the costimulatory potential of the NKG2D/CD28 CCR, we have significantly improved T cell cytolytic activity and proliferation, thus enhancing therapeutic effectiveness against tumors. Consequently, this dual targeting strategy, combining CAR-T with the NKG2D/CD28 receptor, is anticipated to produce a synergistic effect that may enhance the efficacy of CAR-T cell therapy.

In our preclinical models, NKG2D/CD28&CAR-T cells have demonstrated enhanced cytotoxicity against hematological malignancies and solid tumors, especially against tumors expressing low densities of antigens. Incorporating this novel receptor enhances the persistence of CAR-T cells in vivo and substantially increases their capacity to eliminate cancer cells, which is expected to improve the clinical outcomes of CAR-T cell therapy and provide a promising advantage for maintaining long-term anti-tumor effects.

## Materials and methods

### Cell culture

Human cell lines A549, H1299, Nalm6 and K562, as well as the murine B16 cell line, were obtain from American Type Culture Collection (ATCC) and cultured in RPMI 1640 (Sigma-Aldrich, St Louis, CA, USA) with 10% fetal bovine serum (FBS, Sigma-Aldrich). H1299 cells and K562 cells were lentivirally transduced to express human mesothelin and human CD19, respectively, and sorted for different expression densities of target antigens. The Human Embryonic Kidney 293FT cells (ATCC) used for lentiviral packaging were maintained in Dulbecco’s Modified Eagle Medium (DMEM, Sigma-Aldrich) with 10% FBS, GlutaMAX, sodium pyruvate and MEM non-essential amino acids solution (Gibco, Waltham, MA, USA). Luciferase-expressing cells were sorted from wild-type cell lines transfected with lentivirus. All cells were cultured at 37 °C with 5% CO_2_.

### CAR and NKG2D/CD28 constructs

The NKG2D/CD28 chimeric receptors were generated by linking the extracellular domain of NKG2D to the hinge and transmembrane domains of CD28. CD19 CAR constructs used an extracellular scFv as described previously [20], and a CD8 hinge and transmembrane domain, the 4-1BB and CD3ζ signaling domains followed the scFv. The Mesothelin (MSLN) CAR constructs used the 15B6 scFv and have been previously reported [21]. The CAR sequences were ligated to the NKG2D/CD28 chimeric receptor through a 2A self-cleaving peptide (T2A) sequence. CAR and NKG2D/CD28 constructs were cloned into pCDH lentivirus vectors using standard molecular biology techniques.

### Generation lentiviral particles and transduction of T cells

CAR constructs were co-transfected into HEK293FT cells with a mixture of packaging plasmids (pLP1, pLP2, and pVSV-G) at specific ratios, respectively, as described previously [14]. Cell-free supernatants containing lentiviral particles were collected and concentrated by ultracentrifugation at 20 000 g. Healthy donor peripheral blood mononuclear cells (PBMCs) from healthy donors were isolated by Ficoll-Paque (GE Healthcare Life Sciences, Buckinghamshire, UK) density centrifugation. Isolated cells (1 × 10^6^ per well) were stimulated with Dynabeads Human T-Activator CD3/CD28 (Thermo Fisher Scientific, Waltham, MA, USA) in a six-well plate (Corning, New York, USA) in culture medium[X-VIVO 15 (Lonza, Basel, Switzerland) with 50 IU/mL recombinant interleukin-2 (IL-2), 5 ng/mL interleukin-7 and 5 ng/mL interleukin-15 (Perprotech, Rocky Hill, NJ, USA)] for 3 days followed by transduction with lentivirus, and 8 μg/mL polybrene (Sigma–Aldrich) was added to increase the infection efficacy. Transduction efficiency of car and other subsequent experiments were examined 3 days after transduction.

### Antibodies and flow cytometry

Transduction efficiency was measured with a FITC-conjuated antibody toward FLAG (Sigma–Aldrich) for CARs, whereas for the NKG2D/CD28 chimeric receptors, it was measured in the PE channel to detect NKG2D directly (Biolegend, San Diego, CA, USA). The following antibodies were used for flow cytometry staining: BUV395 anti-CD3, PE anti-CD197 (CCR7), APC anti-CD45RA, BUV737 anti-CD279 (PD-1), PE anti-CD366 (TIM-3), AF647 anti-CD223 (LAG-3), PE anti-CD107a, BV421 anti-CD137, APC anti-CD69, PE anti-CD25 (BD Biosciences, Franklin Lakes, NJ, USA). For intracellular marker staining, cells were treated with Cytofix/cytoperm™ Fixation/Permeabilization Kit (BD Biosciences) and followed by staining with AF647 anti-Ki67 antibody (BD Biosciences). For mesothelin and CD19 detection, APC anti-mesothelin and PE anti-CD19 antibody (Biolegend) were used to detect the expression of tumor cell surface antigens. Tumor cells were collected and stained with human LILRB2/CD85d/ILT4-Fc protein (isotype) or NKG2D-Fc protein (R&D Systems, Minneapolis, MN, USA), followed by PE anti-human IgG antibody (Abcam, Cambridge, UK), as previously delineated [14].

### Cytotoxicity assay

To test the lytic effect of target cells, CAR-T cells were co-cultured with carboxyfluorescein succinimidyl ester (CFSE, BD Biosciences)-labeled target tumor cells (5 × 10^4^) at different E: T ratios (1:2–8:1) for 4–6 h. Then, the cells were harvested and stained with 1 μg/mL propidium iodide (PI, BD Biosciences) according to the manufacturer’s guidelines. The fluorescence intensity of the samples was subsequently analyzed using an Accuri C6 flow cytometer (BD Biosciences).

### Cytokine release assays

To determine cytokine production, cell supernatants were harvested 24 h after co-culture with target cells at a specific E: T ratio (2:1) in the absence of exogenous cytokines. Then, the IFN-γ, IL-2, and TNF-α concentrations in the supernatants were quantitatively determine by the enzyme linked immunosorbent assay (ELISA) development kit (R&D Systems) according to the manufacturer’s instructions.

### Functional assay of NKG2D/CD28 CCR

To assess the functionality of the NKG2D/CD28 CCR, non-transduced T cells or T cells transduced with the monomer were stimulated with NKG2D ligand-positive H1299 cells(2:1), anti-CD3 antibody (100 ng/mL, ACRO Biosystems, Beijing, China), anti-CD3 antibody together with H1299 cells, or anti-CD3 and NKG2D-negative B16 cells. After 24 h, cytokine release of IFN-γ was measured using ELISA, and activation markers were analyzed by flow cytometry. After 48 h of stimulation, the expression of the proliferation marker Ki67 was assessed by flow cytometry.

### Phospho-Akt1 assay

CAR T cells (2 × 10^5^) were co-cultured together with target cells (2 × 10^5^) for 15 min at room temperature in X-VIVO 15 basal culture mediums with the recommended amount of BUV395 anti-CD3 antibody. Cells stimulation and staining were performed in 5 ml round-bottom polystyrene FACS (fluorescence activated cell sorting) tubes. At the end of the incubation, cells were washed twice with phosphate buffer (PBS) containing 1% bovine serum albumin (BSA, Sigma Aldrich) and operated on ice whenever possible. The Transcription Factor Buffer Set (BD Biosciences) was used according to the manufacturer’s protocol. After permeabilization and washing, the recommended amount of the AF647 anti-phospho-Akt1 antibody (pS437, BD Biosciences) was added, and the cells were incubated for 30 min at 4 ℃, protected from light. Cells were washed twice with Perm/Wash Buffer and flow cytometry was performed with BD SORP FACS Aria II.

### Repetitive stimulation assay

CAR-T cells were detected and stimulated weekly with tumor cells treated with mitomycin. In briefly, tumor cells (5 × 10^5^) were seeded in a 24-well plate the day before co-culture and pretreated with mitomycin C. CAR-T cells (2.5 × 10^5^) were then added to a final volume of 1 mL of culture with no additional cytokines. For each round of stimulation, T cells were counted and collected, and a fraction used for assay, while the other continued to be stimulated with fresh tumor cells.

### Xenograft model

Female 6-week-old NCG mice (JC Bioscience, Beijing, China) were used to establish the tumor-bearing models under an approved protocol and housed under specific pathogen-free conditions. For the lung cancer in vivo model, a total of 2 × 10^6^ A549-Luc cells or H1299-MSLN^Low^-Luc cells were subcutaneously inoculated into the left flank of the mice. Four days later, 2 × 10^6^ CAR-T or non-transduced T cells were administered via the tail vein. And for the hematologic tumor models, 2 × 10^6^ Nalm6-Luc cells or K562-CD19^Low^-Luc cells were injected intravenously. After seven days, the mice were intravenously injected with 2 × 10^6^ T cells or CAR-T cells. Each mouse was then given an intraperitoneal injection of 6 000 IU IL-2 every other day for three times. Tumor growth was monitored by weekly bioluminescent imaging (BLI) measurements using the IVIS Spectrum CT platform (PerkinElmer) [intraperitoneal injection of 100 μL of D-luciferin Potassium Salt (PerkinElmer)]. Tumor size was also measured with a digital caliper, and the tumor volume was calculated using the formula: volume (mm^3^) = length× width× width /2. Peripheral blood was obtained from the inner canthus and the percentage and subtypes of CAR-T cells were detected by flow cytometry. In addition, mice were weighed every few days. All mice were killed when the animal experiment was terminated.

### Statistical analysis

All data was performed using GraphPad Prism 10.0 (GraphPad Software, San Diego, CA, USA). Data are summarized as mean ± SD or SEM. Statistical significance tests were analyzed by Two-tailed Student’s t-tests or one-way ANOVA with the Bonferroni post-test. From the time of tumor cell injection, the Kaplan-Meier method was used for survival analysis, and the log-rank test was used to compare survival differences between groups. Statistical significance is defined as follows: ns, not significant; *P value < 0.05, **P value < 0.01, ***P value < 0.001.

## Results

### NKG2D/CD28 CCR increases CAR-T cell cytotoxicity against cancer cells

**Fig. 1:**
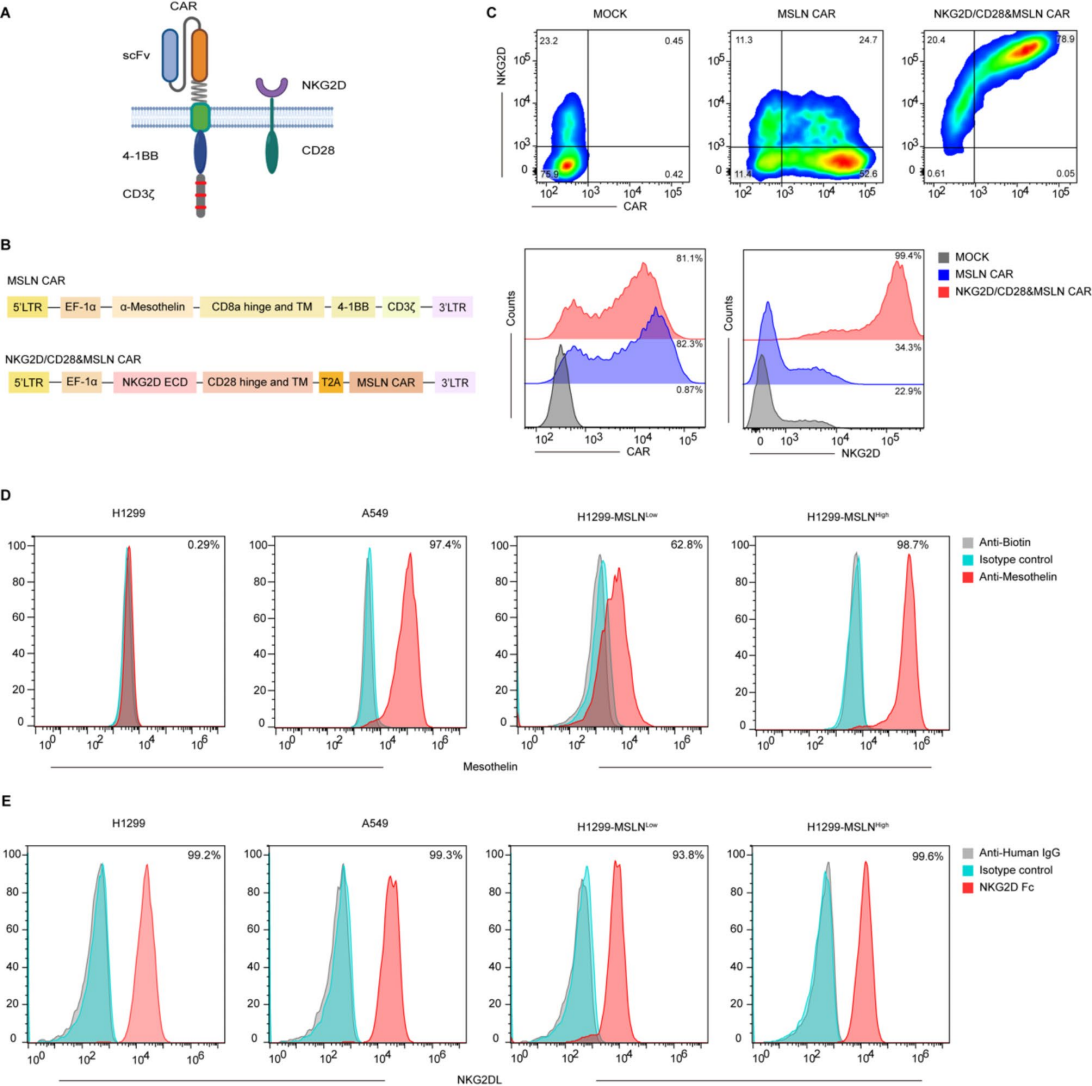
Generation and characterization of NKG2D/CD28&CAR-T cells. **A.** Schematic representation of the structure of CAR-T cells and the NKG2D/CD28 CCR. This panel illustrates the design of the CAR and the NKG2D/CD28, highlighting the components involved in T cell activation and co-stimulation. **B.** Schematic of lentiviral vectors for MSLN CAR and NKG2D/CD28&MSLN CAR. The diagram shows the structure of the lentiviral vectors used to transduce MSLN CAR and NKG2D/CD28&MSLN CAR into T cells, including the relevant genetic elements for expression of the chimeric receptors. **C.** Representative flow cytometry plots showing the expression of CAR and NKG2D in transduced T cells. CAR expression was detected using an anti-FLAG antibody, while NKG2D expression was assessed with an anti-NKG2D antibody, demonstrating the successful transduction and surface expression of the chimeric receptors on T cells. MOCK represents non-transduced T cells. **D.** Flow cytometric analysis of MSLN expression on tumor cells. MSLN expression was analyzed using an anti-MSLN antibody (red histogram), with isotype control (blue histogram) or secondary antibody only (gray histogram). **E.** NKG2DL expression was assessed using recombinant human NKG2D-Fc protein (red histogram), with isotype control (blue histogram) or secondary antibody only (gray histogram), indicating the presence of NKG2DLs on the tumor cells

In this study, we engineered NKG2D/CD28 CCR, to be co-expressed with a traditional second-generation CAR, as illustrated in the schematic figure (Fig. 1A), and sought to evaluate its potential to enhance the antitumor functions of CAR-T cells. The CAR consists of a single-chain variable fragment (scFv) domain for antigen recognition, a CD8a hinge and transmembrane region, and intracellular signaling domains comprising 4-1BB and CD3ζ. The NKG2D/CD28 CCR was constructed using the extracellular ligand-binding domain of NKG2D, connected to the transmembrane and intracellular signaling domain of CD28. To assess the anti-tumor functionality of CAR-T cells expressing this CCR, we constructed two distinct CAR-T cell models: MSLN CAR targeting lung cancer cells (Fig. 1B) and CD19 CAR targeting hematological malignancies (Fig.S1A). Traditional second-generation CARs were used as controls, and experiment groups were then created by linking the CAR to the NKG2D/CD28 receptor via a T2A peptide, allowing for co-expression of both receptors in the same cell. Subsequent experiments were conducted to comparatively analyze the functionality of these engineered CAR-T cells. The rationale for this dual approach was to evaluate the effectiveness of the NKG2D/CD28 receptor across solid and hematological tumors. Flow cytometry analysis demonstrated the presence of both CAR and NKG2D proteins on the T cell membrane (Fig. 1C and Fig.S1B), laying the groundwork for subsequent functional assays.

In addition to the CAR constructs, we also evaluated the functionality of the NKG2D/CD28 CCR when introduced alone into T cells. The NKG2D/CD28 monomer was transduced into T cells, and flow cytometry confirmed high expression of NKG2D (Fig.S2A). We selected NKG2D ligand-positive H1299 cells and NKG2D ligand-negative B16 cells for functional validation, and flow cytometry confirmed the expression of NKG2D ligands on these cell lines (Fig.S2B). After stimulation under different conditions, we measured IFN-γ secretion, Ki67 expression, and activation markers (CD69, CD25, and CD137). While H1299 cells alone did not effectively activate NKG2D/CD28 T cells, the presence of H1299 cells in combination with anti-CD3 antibody stimulation significantly enhanced T cell activation. This was evidenced by increased IFN-γ secretion (Fig.S2C), higher Ki67 expression (Fig.S2D), and elevated activation markers (Fig.S2E). These findings demonstrate that the NKG2D/CD28 CCR in T cells enhances their activation potential, particularly when interacting with NKG2D ligand-positive cells.

To assess the specificity and cytotoxic potential of our NKG2D/CD28&CAR-T cells, we analyzed the expression of target antigens and NKG2DLs across a panel of tumor cell lines. Using flow cytometry with specific antibodies, we verified the presence of MSLN on the A549 lung cancer cell line with a notably high antigen expression density (Fig. 1D). Moreover, we genetically engineered the wild-type H1299 cell line, which lacks endogenous MSLN expression, to stably overexpress MSLN at different levels. We selected cell lines with low and high antigen expression densities for further experiments, termed H1299-MSLN^Low^ and H1299-MSLN^High^, respectively, to mimic the heterogeneity observed in tumors (Fig. 1D and Fig.S1C). We also evaluated the expression of NKG2DL on several cell lines using recombinant NKG2D protein and found high expression levels of NKG2D ligands across these cell lines (Fig. 1E). This finding is consistent with previous studies indicating that NKG2DL expression is upregulated in various tumor types. The high expression of NKG2DL provides an additional target for NKG2D/CD28&CAR-T cells, enhancing their cytotoxic potential against tumor cells with varying antigen densities. Additionally, we utilized the CD19-expressing Nalm6 cell line and generated low and high-density CD19-expressing cell lines by overexpressing CD19 in the CD19-negative K562 cell line (Fig.S1D-E). We also assessed the NKG2DL expression on these cell lines and confirmed their high expression levels (Fig.S1F). This expanded our model to include cell lines with varying densities of CD19 expression, providing a platform for assessing the cytotoxicity of our NKG2D/CD28&CAR-T cells against hematological and solid tumors.

**Fig. 2:**
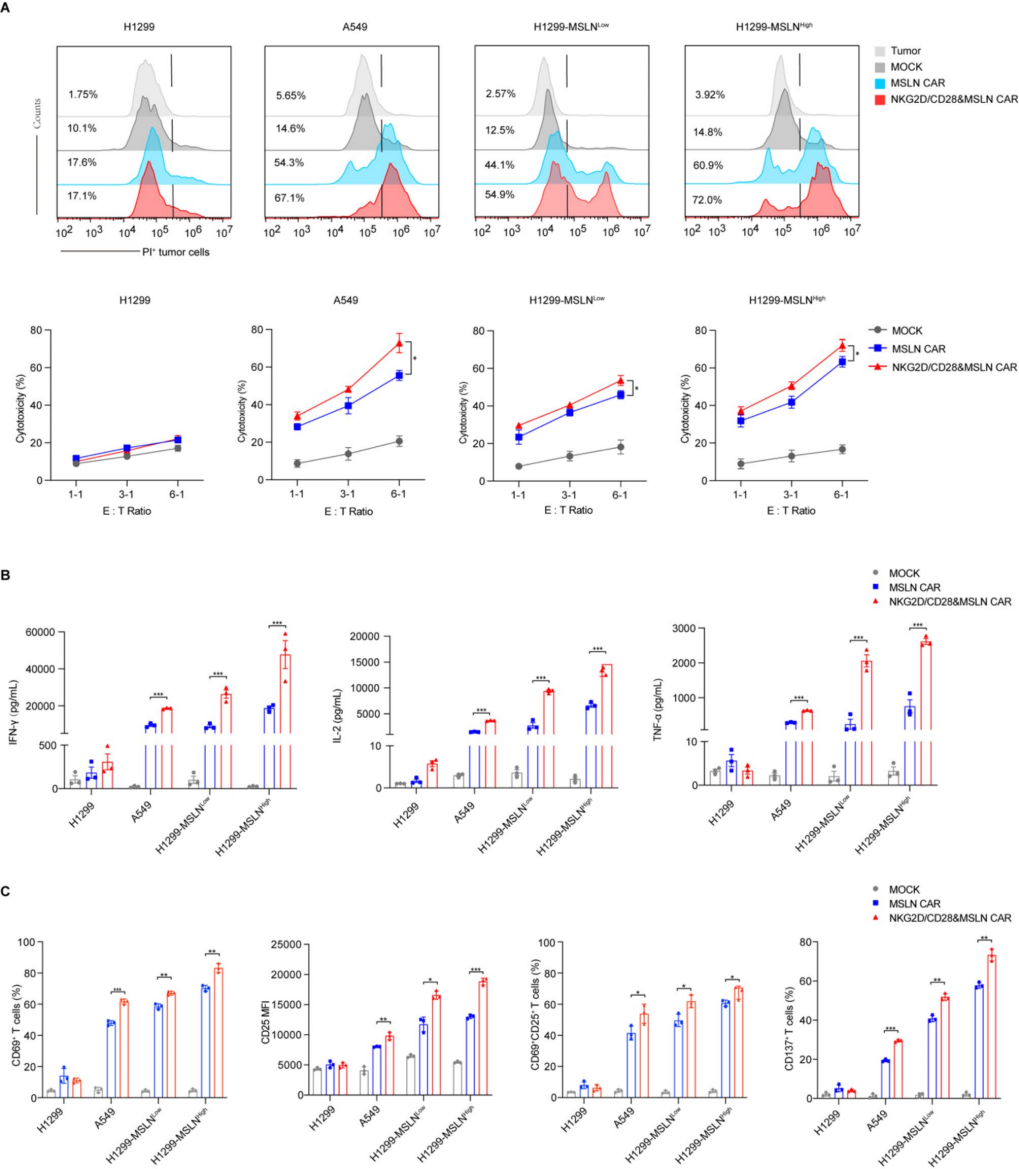
Assessment of cytotoxicity and activation of CAR-T cells against tumor cells. **A.** Cytotoxicity assay against H1299, A549, H1299-MSLN^Low^, and H1299-MSLN^High^ cells. MSLN CAR and NKG2D/CD28&MSLN CAR-T cells were co-cultured with tumor cells at various E: T ratios for 4 h. PI positivity represents the proportion of tumor cells killed after co-culturing with T cells. The figure shows representative flow cytometry histograms and statistical graphs of the cytotoxicity (*n* = 3). **B.** ELISA detection of cytokine secretion in the supernatant after co-culturing MSLN CAR-T cells or NKG2D/CD28&MSLN CAR-T cells with tumor cells at an E: T ratio of 2:1 for 24 h. The levels of IFN-γ, IL-2 and TNF-α in the supernatant were measured to assess the cytokine release from T cells upon interaction with tumor cells. **C.** Expression of T cell activation markers CD69, CD25, and CD137 following co-culturing with tumor cells. The figure displays the flow cytometry analysis of T cell activation markers to evaluate the immune response and activation status of T cells post co-culture with tumor cells. MFI represents mean fluorescence intensity. Statistical significance is defined as follows: *P < 0.05, **P < 0.01, ***P < 0.001

Compared to traditional CAR-T cells, our modified CAR-T cells demonstrated a significant increase in cytotoxicity, effectively targeting tumor cells even at low antigen densities, and there were no tumor killing differences targeting wild-type H1299 cell line and K562 cell line (Fig. 2A and Fig.S3A). In addition to the cytotoxicity assays, we further evaluated the functional impact of NKG2D/CD28&CAR-T cell co-stimulation by measuring cytokine production and activation markers on the CAR-T cells using ELISA assay and flow cytometer. Our results revealed a robust secretion of IFN-γ, TNF-α, and IL-2 by NKG2D/CD28&CAR-T cells, indicative of a potent T cell response (Fig. 2B and Fig.S3B). The increased expression of activation markers CD69, CD25, and CD137 on CAR-T cells after co-culture further confirmed their enhanced responsiveness. (Fig. 2C and Fig.S3C). The expression of CD25 was measured using mean fluorescence intensity (MFI), while the frequency of CD69 and CD137 positive cells was determined, along with the proportion of CD25 and CD69 double-positive cells. The use of MFI for CD25 is particularly appropriate in this context because the presence of IL-2 in the culture medium, can upregulate CD25 expression on the surface of activated T cells. These findings underscore the ability of NKG2D/CD28&CAR-T cells to generate a potent antitumor immune response in vitro.

In summary, co-expressing the NKG2D/CD28 receptor enhanced the cytotoxicity of CAR-engineered T cells against both solid and hematologic malignancies. Our findings suggest that this CCR may improve the efficacy of CAR-T cell therapy, particularly in addressing tumor escape mechanisms associated with low antigen expression. Additionally, the observed increase in cytokine production and T cell activation markers supports the potential of the NKG2D/CD28&CAR-T cell approach in strengthening antitumor immune responses.

### NKG2D/CD28&CAR-T cells show reduced exhaustion and enhanced Naïve phenotype post-stimulation

**Fig. 3:**
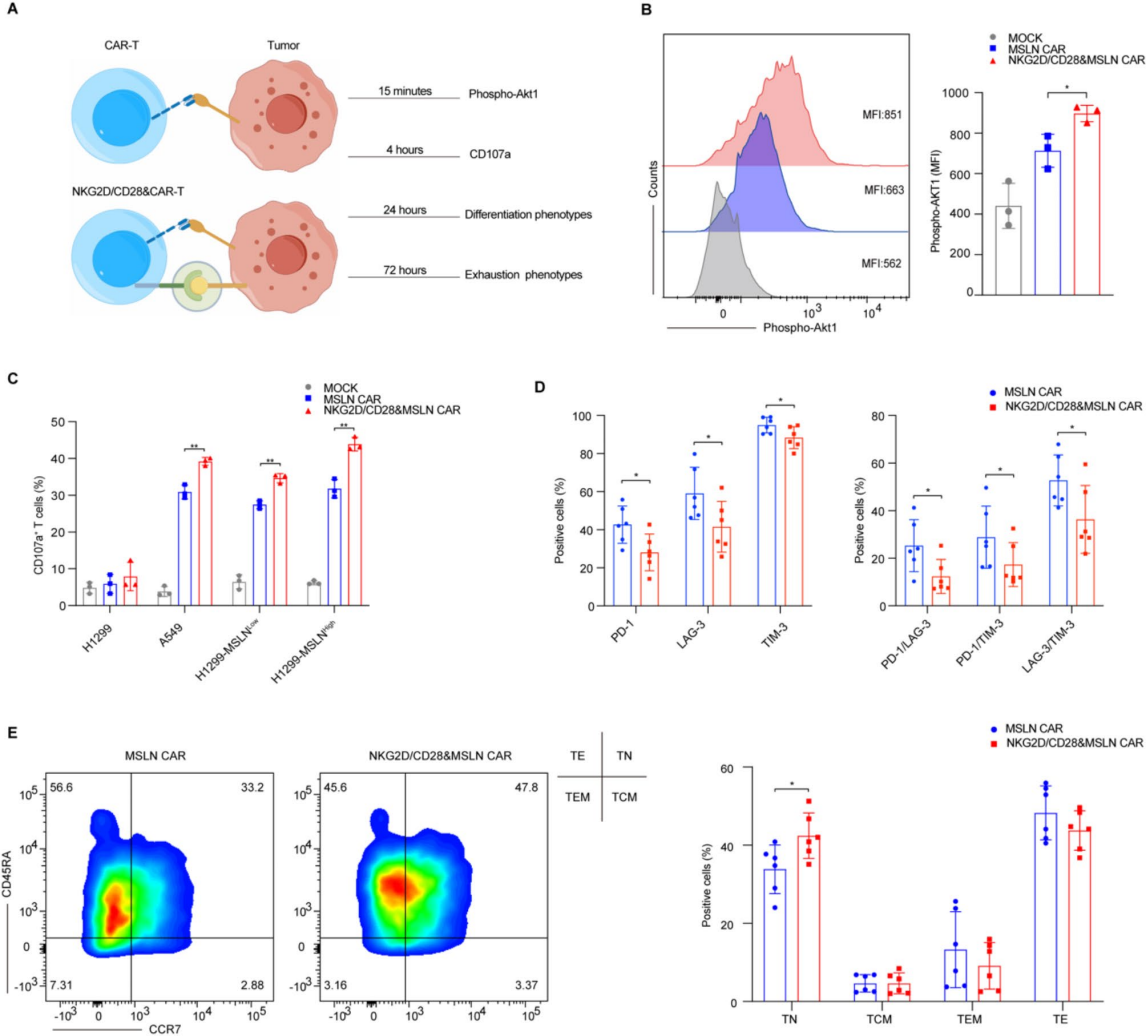
Analysis of CAR-T cell activation and phenotypes following co-culture with tumor cells. **A.** Schematic representation of co-culture of CAR-T cells and tumor cells. CAR-T and NKG2D/CD28&CAR-T cells were co-cultured with tumor cells for various durations to assess differences in T cell phenotypes. The diagram illustrates the experimental setup for the co-culture and subsequent analysis of T cell activation and phenotype. **B.** Flow cytometry analysis of phosphorylated Akt1 in MSLN CAR and NKG2D/CD28&MSLN CAR-T cells after 15 min of co-culture with tumor cells. The figure shows representative histograms and statistical graphs of the MFI of phosphorylated Akt1, indicating the activation status of the T cells upon stimulation with tumor cells. **C.** Flow cytometry detection of CD107a expression in CAR-T cells after 4 h of co-culture with tumor cells. The figure displays the proportion of T cells expressing CD107a, a marker of cytotoxic granule release, indicating the cytotoxic potential of the T cells. **D.** Bar graphs showing the expression of exhaustion markers PD-1, LAG-3, and TIM-3 in MSLN CAR and NKG2D/CD28&MSLN CAR-T cells after 72 h of co-culture with tumor cells. The graphs depict both the expression of single-positive exhaustion markers and the proportion of double-positive cell populations, highlighting the differences in T cell exhaustion between the two CAR-T cell populations. **E.** Flow cytometry analysis of the differentiation phenotype of MSLN CAR and NKG2D/CD28&MSLN CAR-T cells after 24 h of co-culture with tumor cells. The figure includes representative flow cytometry plots and statistical graphs of the T cell differentiation markers, indicating the phenotypic changes in T cells following co-culture with tumor cells. Statistical significance is defined as follows: *P < 0.05, **P < 0.01, ***P < 0.001

A critical aspect of this study was to evaluate how the co-expression of the NKG2D/CD28 CCR impacts the activation and subsequent antitumor functions of CAR-T cells, particularly in relation to the CD28 costimulatory domain known to enhance T cell responses. As previously described, we assessed cytotoxic effects by co-culturing with target cells for 4 h and cytokine secretion after 24 h of co-culture, both of which directly demonstrate the anti-tumor functionality of CAR-T cells in vitro. To further investigate the activation and subsequent anti-tumor effects of NKG2D/CD28&CAR-T cells, an antigen stimulation model was established at multiple time points for a comprehensive assessment (Fig. 3A).

The 15-minute time point was selected to capture the initial phase of T cell activation, providing insight into the rapid signaling events triggered by antigen recognition. This early assessment enabled the measurement of Akt-1 phosphorylation (p-Akt1), a key molecule of the PI3K/Akt signaling pathway, which plays a crucial role in T cell activation and survival. The 4-hour time point was chosen to evaluate the expression of cytotoxic markers such as CD107a, which indicates degranulation capacity and the ability of CAR-T cells to mediate direct tumor cell killing. This time frame represents the effector phase of T cell responses, during which cytotoxic granules are released toward target cells. At 24 h, T cell differentiation phenotypes were analyzed to assess early activation states, while exhaustion markers were examined at 72 h to evaluate long-term functionality. This later time point is particularly relevant as it reflects potential exhaustion, a key factor in determining CAR-T cell persistence and therapeutic efficacy. Together, these time points provide a comprehensive assessment of NKG2D/CD28&CAR-T cell activation, cytotoxicity, differentiation, and persistence.

In our model, T cells were stimulated once with target-expressing tumor cells, and p-Akt1 levels were assessed via flow cytometry after 15 min to evaluate downstream signaling activation. NKG2D/CD28&CAR-T cells demonstrated elevated p-Akt1 levels, indicating stronger initial signaling compared to traditional CAR-T cells (Fig. 3B and Fig.S4A). This early activation is crucial because it can impact the T cells’ subsequent functional responses. After 4 h of stimulation, CD107a expression, a critical marker of degranulation and cytotoxic potential, was measured. NKG2D/CD28&CAR-T cells exhibited higher CD107a expression, suggesting enhanced cytotoxic activity against target cells (Fig. 3C and Fig.S4B).

Following a 24-hour co-culture with target cells, the differentiation phenotype of the T cells was assessed. The results showed that NKG2D/CD28&CAR-T cells exhibited a less differentiated phenotype compared to traditional CAR-T cells, with a higher proportion of cells expressing markers associated with a naïve state, such as CD45RA^+^and CCR7^+^(Fig. 3E and Fig.S4C). T cell differentiation is commonly assessed by the expression of CD45RA and CCR7, where CD45RA distinguishes naïve from memory T cells, with naïve T cells exhibiting high expression levels. CCR7 is involved in the migration of T cells to lymph nodes, a characteristic behavior of naïve T cells. The co-expression of CD45RA and CCR7 indicates a less differentiated state [22] and suggests that NKG2D/CD28&CAR-T cells possess greater proliferative potential, which is favorable for long-term persistence and sustained functionality in vivo.

After 72 h of co-culture, the exhaustion phenotype was evaluated based on the expression of PD-1, LAG-3, and TIM-3. NKG2D/CD28&CAR-T cells exhibited lower levels of all three exhaustion markers compared to traditional CAR-T cells, indicating a potentially less exhausted state (Fig. 3D and Fig.S4D). Additionally, the proportion of double-positive cells co-expressing PD-1 and LAG-3, PD-1 and TIM-3, or LAG-3 and TIM-3 was lower in NKG2D/CD28&CAR-T cells. These findings suggest that the reduced expression of exhaustion markers may contribute to the prolonged persistence and potential functional advantages of NKG2D/CD28&CAR-T cells in tumor clearance.

In conclusion, our findings suggest that NKG2D/CD28&CAR-T cells exhibit increased Akt phosphorylation and higher CD107a expression following antigen stimulation, indicating enhanced signaling and cytotoxic potential. Moreover, these cells display a less differentiated and less exhausted phenotype compared to traditional CAR-T cells. While these observations suggest potential advantages of NKG2D/CD28&CAR-T cells, further studies are required to evaluate their impact on CAR-T cell persistence and tumor clearance in therapeutic settings.

### Sustained anti-tumor activity of NKG2D/CD28&CAR-T cells in chronic antigen stimulation

**Fig. 4:**
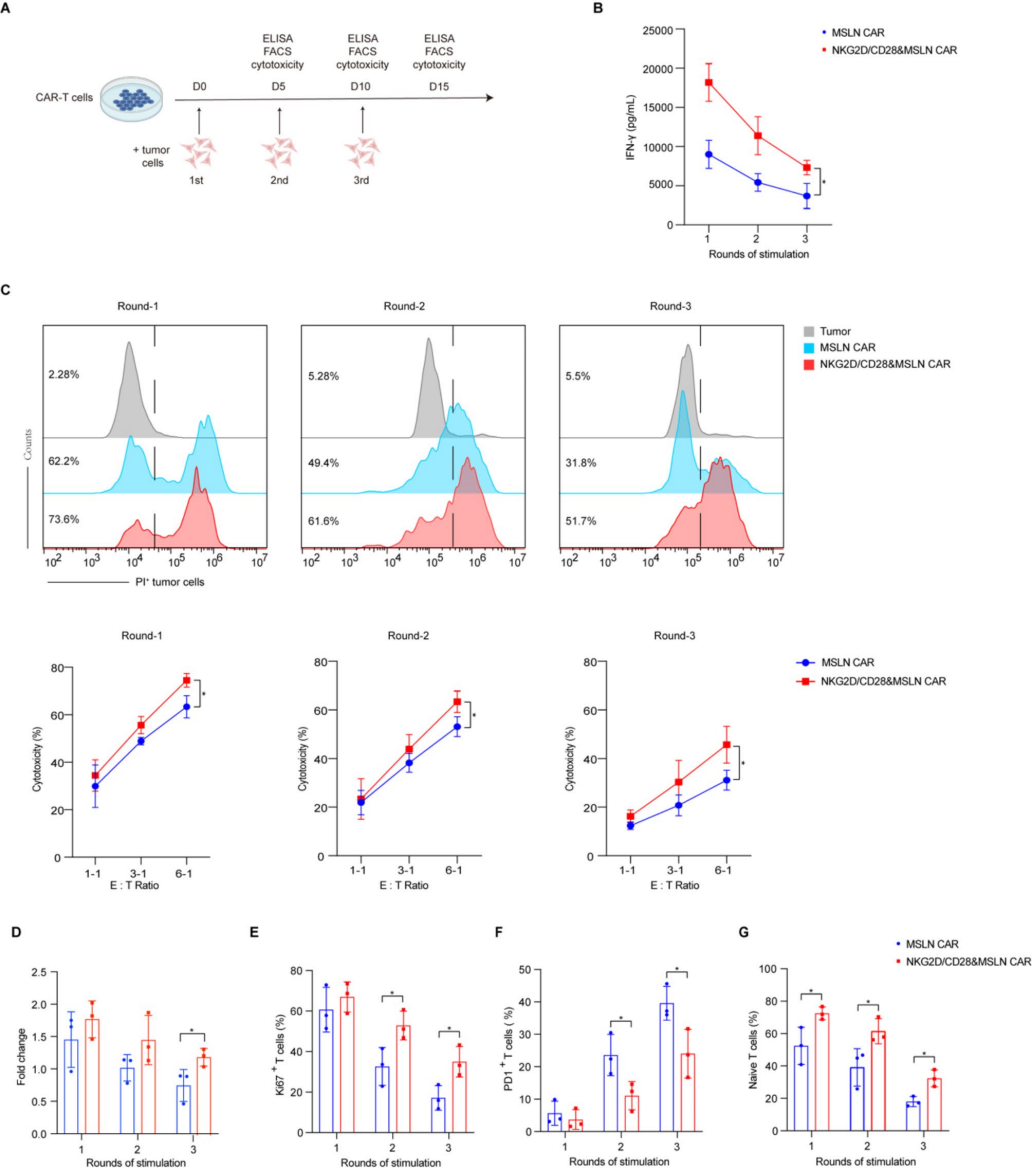
Chronic stimulation of CAR-T cells with mitomycin C-treated tumor cells and subsequent analysis. **A.** Schematic illustration of the chronic stimulation model using mitomycin C-treated tumor cells. CAR-T and NKG2D/CD28&CAR-T cells are stimulated with tumor cells in a cycle of 5 days per round. This panel depicts the experimental design for chronic antigen stimulation and subsequent assessment of T cell responses. **B.** ELISA measurement of IFN-γ secretion by MSLN CAR and NKG2D/CD28&MSLN CAR-T cells following repeated stimulation with tumor cells. The levels of IFN-γ in the supernatant are determined after each 5-day stimulation cycle, indicating the cytokine production by T cells in response to sustained antigen exposure (*n* = 3). **C.** Cytotoxicity assay of CAR-T cells against tumor cells using a gradient of E: T ratios after each round of antigen stimulation. Representative flow cytometry histograms and statistical graphs are presented, showing the capacity of T cells to kill tumor cells following chronic stimulation (*n* = 3). **D.** Counting of viable CAR-T cells after each round of antigen stimulation. The figure shows the proliferation of CAR-T cells, indicating their expansion following repeated exposure to tumor antigens. **E.** Flow cytometry detection of Ki67 expression in CAR-T cells following antigen stimulation. The expression of Ki67, a marker of cell proliferation, is assessed to evaluate the proliferative response of T cells to chronic antigen stimulation. **F.** Flow cytometry detection of exhaustion marker PD-1 on CAR-T cells following antigen stimulation. The figure shows the expression of PD-1, a key marker of T cell exhaustion, to determine the impact of chronic stimulation on T cell fatigue. **G.** Flow cytometry analysis of the proportion of naïve T cells within the CAR-T cell population following antigen stimulation. The figure displays the percentage of naïve T cells, indicating the preservation of a youthful phenotype in the T cell population. Statistical significance is defined as follows: *P < 0.05, **P < 0.01, ***P < 0.001

Chronic antigen exposure can lead to T cell exhaustion, characterized by impaired self-renewal and reduced effector function. This phenomenon is particularly relevant in the context of tumor-infiltrating T cells, which often exhibit a terminally exhausted phenotype due to persistent antigen stimulation. To assess the impact of chronic antigen exposure on the anti-tumor capabilities and phenotypes of CAR-T cells, we established a model involving repeated stimulations with target cells expressing low densities of antigens (Fig. 4A). Specifically, we used H1299-MSLN^Low^ and K562-CD19^Low^ cell lines to chronically stimulate MSLN CAR and CD19 CAR-T cells, respectively. This model was designed to simulate the continuous antigen exposure that CAR-T cells may encounter in a tumor environment, which is crucial for understanding the dynamics of T cell exhaustion and the potential longevity of CAR-T cell therapies. The significance of using target cells with low antigen densities lies in the heterogeneity often observed in tumors, where cells expressing high-density antigens are more likely to be targeted and eliminated by CAR-T cells, allowing cells with lower antigen expression to persist [23]. By employing target cells that exhibit low antigen densities, our model more accurately simulates the conditions that CAR-T cells are likely to encounter during in vivo therapy, accounting for the repeated stimulation by long-lived tumor cells with low-density antigens. This approach provides a more realistic assessment of the CAR-T cells’ antitumor capabilities and their resistance to fatigue, reflecting the need to understand and overcome the challenges posed by the persistence of low-density antigen-expressing tumor cells in the context of CAR-T cell therapy.

In our model, CAR-T cells underwent three rounds of antigen stimulation, each lasting for 5 days. Following each round of stimulation, a portion of the activated CAR-T cells was co-cultured with target cells to evaluate their anti-tumor functionality, including the secretion of the cytokine IFN-γ and cytotoxicity assays. We also assessed the proliferative capacity of the CAR-T cells, which is critical for the effectiveness of adoptive T cell therapy. Additionally, we utilized flow cytometry to analyze the cells’ differentiation and exhaustion phenotypes, providing a comprehensive assessment of how chronic antigen exposure influences CAR-T cells function. This approach facilitated a detailed comparison of NKG2D/CD28&CAR-T cells and traditional CAR-T cells in response to prolonged antigen stimulation.

Our findings showed that although IFN-γ secretion decreased over successive stimulations, NKG2D/CD28&CAR-T cells consistently produced higher IFN-γ levels than the control traditional CAR-T cells (Fig. 4B and Fig.S5A). This indicates that NKG2D/CD28&CAR-T cells maintained relatively higher cytokine production despite chronic antigen exposure. Similarly, cytotoxicity assays revealed a decline in the lytic activity of both CAR-T cell types over time, with NKG2D/CD28&CAR-T cells exhibiting comparatively greater cytotoxic capacity than traditional CAR-T cells (Fig. 4C and Fig.S5B). These results suggest that the co-expression of NKG2D/CD28 may contribute to maintaining CAR-T cell functionality under conditions mimicking the tumor microenvironment.

During the chronic antigen stimulation process, cell numbers were recorded after each round of stimulation, and the fold difference relative to the initial count was calculated. NKG2D/CD28&CAR-T cells exhibited a trend toward greater proliferative capacity than control CAR-T cells, with a significant difference in cell expansion emerging after the third round of stimulation (Fig. 4D and Fig.S5C). To explore this further, we evaluated Ki67 expression, a marker of cellular proliferation, after each round of stimulation. NKG2D/CD28&CAR-T cells showed significantly higher Ki67 expression after the second and third rounds of antigen stimulation, suggesting a potential advantage in proliferative capacity (Fig. 4E and Fig.S5D). Additionally, we analyzed the differentiation phenotypes and PD-1 expression levels of CAR-T cells following each round of antigen stimulation. Aligning with prior observations, NKG2D/CD28&CAR-T cells exhibited lower PD-1 expression levels (Fig. 4F and Fig.S5E) and maintained a higher proportion of naïve T cells (Fig. 4G and Fig.S5F) compared to control CAR-T cells, even after multiple rounds of antigen stimulation.

In summary, our study suggests that NKG2D/CD28&CAR-T cells maintain anti-tumor functionality under chronic antigen stimulation in both MSLN and CD19 CAR-T models. This is reflected in sustained IFN-γ secretion, greater cytotoxic potential, higher Ki67 expression, a higher proportion of naïve T cells, and lower PD-1 expression. These findings indicate that NKG2D/CD28 co-stimulation may help preserve CAR-T cell function under prolonged antigen exposure, warranting further investigation in cancer immunotherapy.

### Superior anti-tumor efficacy and durability of NKG2D/CD28&CAR-T cells in vivo

To evaluate the in vivo antitumor efficacy of NKG2D/CD28&CAR-T cells, we established mouse tumor models using fluorescently-labeled cell lines for both solid and hematological tumors. These models included parental A549 and Nalm6 cell lines, which endogenously express the target antigens, as well as engineered low-antigen-expressing derivatives H1299-MSLN^Low^ and K562-CD19^Low^. This design enabled the assessment of NKG2D/CD28&CAR-T cells against tumors with varying antigen densities, providing a comprehensive assessment of their therapeutic potential in a preclinical setting.

**Fig. 5:**
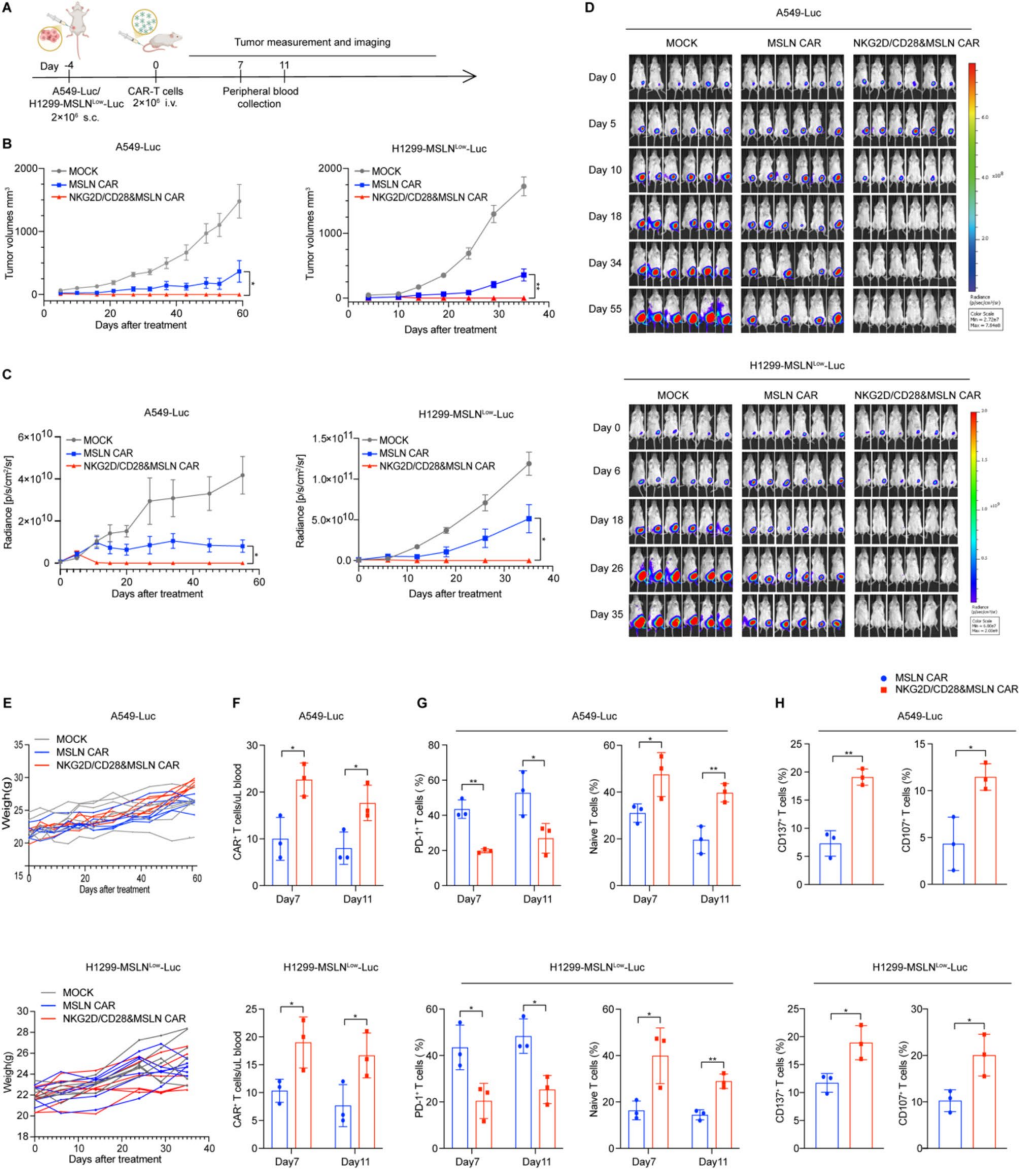
Establishment and analysis of xenograft solid tumor models and evaluation of CAR-T cell therapy. **A.** Schematic illustration of the xenograft tumor model establishment process. A total of 2 × 10^6^ A549-Luc/H1299-MSLN^Low^-Luc cells were subcutaneously implanted, and non-transduced T cells, MSLN CAR, and NKG2D/CD28&MSLN CAR-T cells were administered via tail vein injection 4 days later. Tumor burden was regularly monitored using an in vivo imaging system, and tumor size was measured with calipers. Peripheral blood was collected from the mice at days 7 and 11 post-T cell infusion for flow cytometry analysis. **B.** Changes in tumor burden measured by caliper. The graph shows the physical dimensions of the tumors over time, as measured by external calipers, indicating the growth or regression of the tumors in response to T cell therapy. **C.** Quantification of average tumor burden in mice using BLI, expressed in photons per second per square centimeter per steradian (ph/s/cm^2^/sr) (*n* = 6 mice per group). The graph represents the average tumor burden across all mice in each group, as measured by the bioluminescent signal. **D.** Bioluminescence imaging (BLI) snapshots at multiple time points during the experiment, with pixel intensity represented in color. The images illustrate the tumor burden in the mice over time, as visualized by the bioluminescent signal emitted by the A549-Luc cells and H1299-MSLN^Low^-Luc cells. **E.** Body weight changes of mice post-T cell infusion. The graph tracks the weight changes of the mice, which can indicate the overall health and potential side effects of the T cell treatment. **F.** Flow cytometry detection of the percentage of CAR^+^ T cells in the peripheral blood of mice at days 7 and 11 post-T cell infusion. The histogram displays the proportion of CAR-T cells in the peripheral blood, demonstrating the persistence and circulation of the infused T cells. **G.** Histograms showing the percentage of PD-1^+^ and naïve T cells in the peripheral blood of mice post-T cell infusion. The graphs indicate the level of exhaustion and the presence of naïve T cells in the peripheral blood, which can reflect the activation status and potential for long-term efficacy of the T cell therapy. **H.** Flow cytometry detection of CD137 and CD107a expression on peripheral blood T cells at day 7 post-tail vein injection of T cells. The graphs represent the activation markers CD137 and CD107a, which are indicative of the cytotoxic potential and activation status of the T cells post-infusion. Statistical significance is defined as follows: *P < 0.05, **P < 0.01, ***P < 0.001

We performed subcutaneous inoculations for the solid tumor model and tail vein injections for the hematologic tumor model. Following inoculation, CAR-T cells were administered via tail vein injection on day 4. Subsequently, we conducted regular in vivo fluorescence imaging and measured tumor size with calipers (Fig. 5A). In both the A549 and H1299-MSLN^Low^ tumor models, MSLN CAR-T cells and NKG2D/CD28&MSLN CAR-T cells demonstrated significant tumor regression compared to the non-transduced T cell control group, as evidenced by reduced tumor volumes and in vivo imaging. Notably, NKG2D/CD28& MSLN CAR-T cell group showed enhanced tumor suppression, with a significant difference observed when compared to the traditional MSLN CAR-T cell group (Fig. 5B-D). In the A549 tumor model, while only two mice in the traditional MSLN CAR-T group exhibited near-complete tumor clearance, all mice in the NKG2D/CD28& MSLN CAR-T group achieved full tumor clearance. In the H1299-MSLN^Low^ tumor model, which presents a low-density antigen challenge, traditional MSLN CAR-T cells showed some tumor suppressive effects but these were less pronounced than in the A549 model, and no complete tumor clearance was observed. In contrast, NKG2D/CD28& MSLN CAR-T cells maintained robust antitumor activity in this low-density antigen context, leading to complete tumor clearance in all mice.

We monitored mouse weight to assess potential toxic side effects of CAR-T cell therapy and found no significant weight loss, indicating the absence of severe toxic effects (Fig. 5E). To evaluate the persistence and phenotype of CAR-T cells post-infusion, peripheral blood samples were collected on days 7 and 11 for flow cytometry analysis. Consistently across two tumor models, the NKG2D/CD28&MSLN CAR-T group exhibited a higher CAR-T cell count per microliter of blood at both time points (Fig. 5F), with a greater proportion of naïve T cells and lower PD-1 expression compared to the traditional CAR-T group (Fig. 5G). These findings suggest that NKG2D/CD28&MSLN CAR-T cells may persist longer in circulation. Additionally, higher expression levels of the activation marker CD137 and cytotoxicity marker CD107a were detected in this group on day 7, indicating an enhanced activation state and cytotoxic potential (Fig. 5H).

**Fig. 6:**
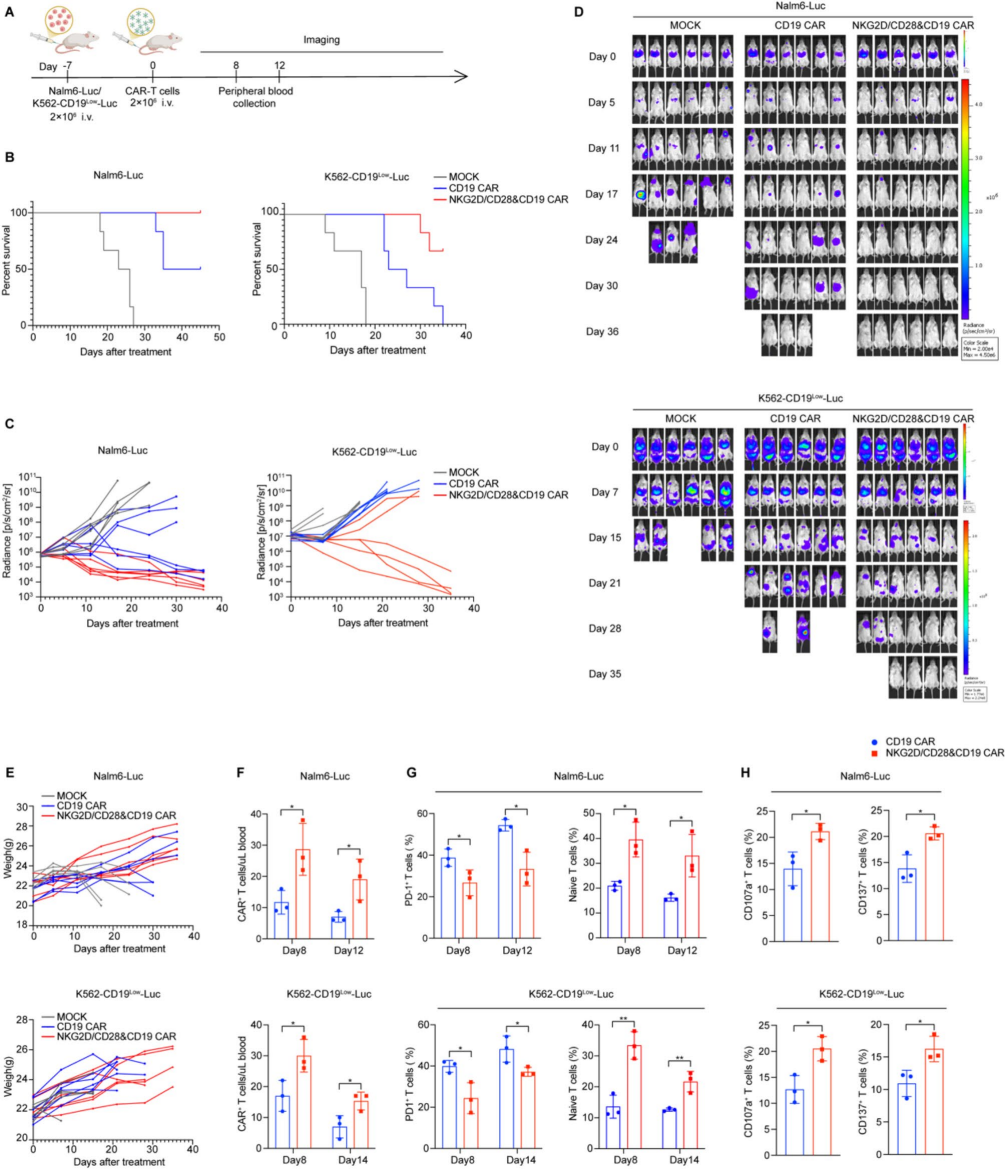
Comprehensive analysis of hematological tumor xenograft model and CAR-T cell therapy efficacy. **A.** Schematic diagram of the process for establishing a murine model of hematological tumors. 2 × 10^6^ Nalm6-Luc and K562-CD19^Low^-Luc cell lines were injected via the tail vein, followed by the tail vein infusion of T cells one week later. Tumor growth was regularly monitored using in vivo imaging, and mouse body weights were recorded. Additionally, mouse periorbital blood was collected on days 8 and 12 post-T cell infusion for flow cytometry analysis (*n* = 6 mice per group). **B.** Survival rates of mice in both models are depicted. **C.** The average tumor burden of mice over time, with tumor burden represented in photons per second per square centimeter per steradian (ph/s/cm^2^/sr). **D.** Illustrative images captured at various time points, where color intensity corresponds to pixel values. **E.** Temporal variations in murine body mass measurements. **F.** Flow cytometry detection of the proportion of CAR^+^ T cells in peripheral blood of mice on days 8 and 12 post-T cell infusion. **G.** The proportion of PD-1^+^ T cells and naïve T cells in the peripheral blood of mice post-T cell infusion. **H.** Expression of CD137 and CD107a in T cells from the peripheral blood of mice on day 8 post-T cell infusion. Statistical significance is defined as follows: *P < 0.05, **P < 0.01, ***P < 0.001

For hematological tumor xenograft models, tumors were established by tail vein injection of tumor cells, followed by tail vein infusion of CAR-T cells one week later. Tumor growth was monitored through regular in vivo fluorescence imaging (Fig. 6A). In both the Nalm6 and K562-CD19^Low^ tumor models, the NKG2D/CD28&CD19 CAR-T cell group demonstrated prolonged survival and greater tumor growth inhibition compared to the traditional CD19 CAR-T cells (Fig. 6B-D). In the Nalm6 model, more mice in the NKG2D/CD28&CD19 CAR-T cell group achieved complete tumor clearance compared to the traditional CD19 CAR-T cell group. In the K562-CD19^Low^ model, which presents low-density antigens, the traditional CAR-T cells extended survival and partially suppressed tumor growth but did not achieve complete clearance. In contrast, NKG2D/CD28&CD19 CAR-T cells demonstrated stronger tumor control, with complete clearance observed in a subset of treated mice.

Consistent with the solid tumor models, mouse body weight was monitored to assess potential toxic side effects, and no significant weight loss was observed (Fig. 6E). Additionally, we analyzed the CAR-T cell proportion in mouse peripheral blood, PD-1 expression, the proportion of naïve T cells, and the expression levels of CD107a and CD137. The results showed that NKG2D/CD28&CD19 CAR-T cells had a higher proportion in peripheral blood compared to traditional CAR-T cells (Fig. 6F). These cells also exhibited a greater proportion of naïve T cells and lower PD-1 expression levels (Fig. 6G). Moreover, the expression levels of CD107a and CD137 were elevated in the NKG2D/CD28&CD19 CAR-T cell group, suggesting enhanced cytotoxic potential and activation. (Fig. 6H).

These findings were observed in both MSLN and CD19 CAR-T cell models, indicating that NKG2D/CD28&CAR-T cells exhibit sustained antitumor activity and persistence in vivo. This suggests that the NKG2D/CD28 modification may help maintain antitumor responses and mitigate T cell exhaustion, supporting its potential for further exploration in cancer immunotherapy.

## Discussion

The field of CAR-T cell therapy has made significant strides in treating hematological malignancies, yet it faces challenges that limit its broader application, particularly in solid tumors [24]. One of the primary obstacles is the limited persistence of CAR-T cells within the host, which can lead to tumor recurrence and reduced therapeutic efficacy [25]. Additionally, the downregulation of target antigen expression on tumor cells can render CAR-T cells ineffective over time [26]. To address these limitations, recent research has proposed the co-expression of CCRs with CARs to enhance the efficacy of T cell responses [9–12]. For instance, combining BCMA- or CD19-targeted CARs with CD38-directed CCRs has been reported to improve tumor cell killing both in vitro and in vivo, even in the context of low antigen density [9]. Integrating CCRs with CARs can lower the activation threshold of T cells, promote their expansion, and potentially enhance their overall antitumor activity, which may lead to improved clinical outcomes in CAR-T therapy. In our previous research, we explored the design of PD-1/CD28 immune stimulatory fusion proteins to enhance CAR-T cell functionality and persistence by converting inhibitory signals into co-stimulatory signals [19]. Building on this concept, the present study introduced NKG2D/CD28 as a CCR in CAR-T cells. Our findings suggest that co-expressing NKG2D/CD28 with CAR enhances the antitumor activity and persistence of CAR-T cells, and this dual-targeting strategy is designed to enhance the activity and breadth of antitumor both in vivo and in *vitro*.

Research has underscored that the selection of CCRs for CAR-T cell therapy has specific requirements, notably the need for the targeted antigen to be highly expressed and the receptor to have a high affinity for its ligand [9]. NKG2D plays a significant role in tumor antigen recognition, acting as an activating receptor expressed on NK cells and certain T cell subsets. Its ligands are often upregulated in transformed or stressed cells, enabling immune recognition and activation [27]. In comparison to other NK cell surface receptors like NKG2C, NKp30 and KIRs, NKG2D has a broader ligand recognition profile making it an attractive candidate for immunotherapeutic applications [28].

The therapeutic applications of NKG2D in cancer immunotherapy are diverse and include bispecific antibodies and CAR-T cells [29]. For instance, a bispecific antibody, HER2-CRB, which targets HER2 and contains an NKG2D agonist arm, has been shown to enhance the effector function of NK and CD8^+^ T cells in preclinical models [30]. While NKG2D-CAR T cells have shown impressive benefits in hematological cancer patients, challenges remain in the context of solid tumors, including poor persistence of T cells and immunosuppression [2, 6, 31]. To address these challenges, previous studies have explored modifications to NKG2D-based CAR constructs, aiming to improve tumor targeting, cytokine secretion, and CAR-T cell persistence [14]. Given the well-established role of NKG2D in recognizing tumor-associated antigens, further research into its immunotherapeutic applications may contribute to advancing CAR-T cell strategies.

The co-expression of the NKG2D/CD28 CCR in CAR-T cells offers a dual-activation strategic advantage by enhancing the anti-tumor functions and maintaining a youthful phenotype. When combined with the CD28 co-stimulatory signal, this configuration not only boosts the activation of T cells but also modulates their proliferation, cytokine production, and cytotoxic potential [17, 18]. The observed increase in p-Akt levels in NKG2D/CD28&CAR-T cells further suggests that this receptor contributes to enhanced T cell signaling, which are critical for effective antitumor responses. The internal mechanisms that allow NKG2D/CD28&CAR-T cells to sustain their anti-tumor activity and youthfulness are multifaceted and may involve the intricate interplay between co-stimulatory signals and intracellular pathways. Based on our findings, future research should focus on a more detailed understanding of these mechanisms, and further in-depth studies are warranted to elucidate specific molecular pathways and cellular interactions that contribute to the enhanced functionality of NKG2D/CD28&CAR-T cells. In addition, we can further improve our CAR-T therapy by understanding the metabolic requirements of these cells and how they differ from traditional CAR-T cells, ensuring their safety, effectiveness, and potential to provide lasting benefits in cancer treatment.

While concerns have been raised regarding the potential off-target toxicity to healthy cells under stress conditions due to the broad expression of NKG2DLs [16], our study provides reassurance on the specificity of NKG2D/CD28&CAR-T cells. We have demonstrated that these engineered cells exhibit minimal cytotoxicity against CAR target-negative H1299 and K562 cell lines, indicating a lack of off-target effects. Although NKG2D ligands may not be entirely tumor-specific, their widespread presence in tumors and the tumor microenvironment can effectively activate the NKG2D receptor, which is why we chose NKG2D as a CCR rather than as a direct CAR target. Consequently, our design retains the specificity of CAR-T cell therapy, reducing the risk of unintended damage to healthy tissues, while also enhancing the antitumor activity and persistence of CAR-T cells through dual signaling. Furthermore, given the widespread expression of NKG2D ligands across various tumor types, the NKG2D/CD28 CCR exhibits broad applicability and has the potential to enhance antitumor responses when utilized in conjunction with a diverse array of CAR constructs.

Despite the promising findings, our study has several limitations that warrant acknowledgment. We primarily focused on the CD28 and 4-1BB costimulatory domains, both of which are well-established in enhancing CAR-T cell function [11, 32, 33]. However, we did not explore other signaling domains or various domain combinations that could potentially yield superior outcomes. Beyond CD28, other co-stimulatory molecules like ICOS, CD27, and OX40 are being investigated for their potential to enhance CAR-T cell function. These molecules offer unique signaling profiles that can influence T cell activation, proliferation, and memory formation [34, 35]. For instance, CD27-based CAR T cells have demonstrated higher antitumor activity and increased survival in tumor-bearing mouse models, upregulating IL-7Rα expression while downregulating PD-1 expression [36]. ICOS and OX40 tandem co-stimulation has been shown to enhance CAR T-cell cytotoxicity and promote a T-cell persistence phenotype [37]. Each of these co-stimulatory domains may provide different advantages and disadvantages compared to CD28. Therefore, the optimal choice may depend on the specific treatment background and the nature of the targeted tumor.

Furthermore, our study’s evaluation of antigen densities was restricted to levels above established thresholds, with no examination of densities below these limits. This is an important consideration, as antigen density can influence the recognition and activation of CAR-T cells, with densities falling below certain thresholds potentially failing to elicit a response from CAR-T cells [38]. Therefore, future studies should investigate the cytotoxic activity of NKG2D/CD28&CAR-T cells across a lower spectrum of antigen densities. This approach would provide a deeper understanding of how these cells perform against tumors with minimal antigen expression. Concurrently, designing CAR-T cells that can discern low-density antigens while retaining the capacity to distinguish between malignant and healthy cells is crucial for ensuring a safe and effective therapeutic window [7, 39].

Besides the previously discussed points, another limitation of this study is that it primarily relies on cell line-based models, which, while providing valuable insights into the functional properties of NKG2D/CD28&CAR-T cells, may not fully recapitulate the complexity of human tumors. Future studies should incorporate patient-derived tumor samples or organoid models to better assess the therapeutic potential and translational applicability of this approach. Additionally, the immunosuppressive tumor microenvironment remains a significant challenge for CAR-T cell therapy, particularly in solid tumors [24]. Factors such as regulatory T cells, myeloid-derived suppressor cells, and immunosuppressive cytokines can inhibit CAR-T cell function and persistence. While our design aims to enhance T cell activation and persistence, further strategies, including metabolic reprogramming, gene editing to enhance resistance to immunosuppression, or combination therapies with immune checkpoint inhibitors, should be explored to improve the efficacy of NKG2D/CD28&CAR-T cells in overcoming these challenges.

## Conclusions

Our results suggest that the NKG2D/CD28 CCR, when combined with a CAR, could enhance immune therapy outcomes for malignant tumors such as B-cell lymphoma and lung cancer. The observed improvements in cytotoxicity, reduced exhaustion, and enhanced in vivo persistence of NKG2D/CD28&CAR-T cells highlight their potential as a valuable approach in cancer immunotherapy. This study provides a rationale for further investigation into the application of NKG2D/CD28&CAR-T cells, offering a promising approach to address the challenges of antigen expression downregulation and CAR-T cell persistence.

## Electronic supplementary material

Below is the link to the electronic supplementary material.

Supplementary Material 1

## Data Availability

No datasets were generated or analysed during the current study.

## Abbreviations

CAR: Chimeric antigen receptor
CCR: Chimeric co-stimulatory receptor
CFSE: Carboxyfluorescein succinimidyl ester
ELISA: Enzyme-linked immunosorbent assay
IFN-γ: Interferon-γ
IL-2: Interleukin-2
MSLN: Mesothelin
NKG2D: Natural killer group 2 member D
NKG2DL: NKG2D ligand
PBMC: Peripheral blood mononuclear cell
PI: Propidium iodide
TNF-α: Tumor necrosis factor-α
