# Supplementary material for: NKG2D/CD28 chimeric receptor boosts cytotoxicity and durability of CAR-T cells for solid and hematological tumors
Source: Exp Hematol Oncol. 2025 Apr 3;14:52. doi: 10.1186/s40164-025-00646-3 (PMC11967049; doi:10.1186/s40164-025-00646-3)
Supplement: Supplementary file 1 — Supplementary Material 1 [file 40164_2025_646_MOESM1_ESM.docx]

**Supplementary Figures**

**
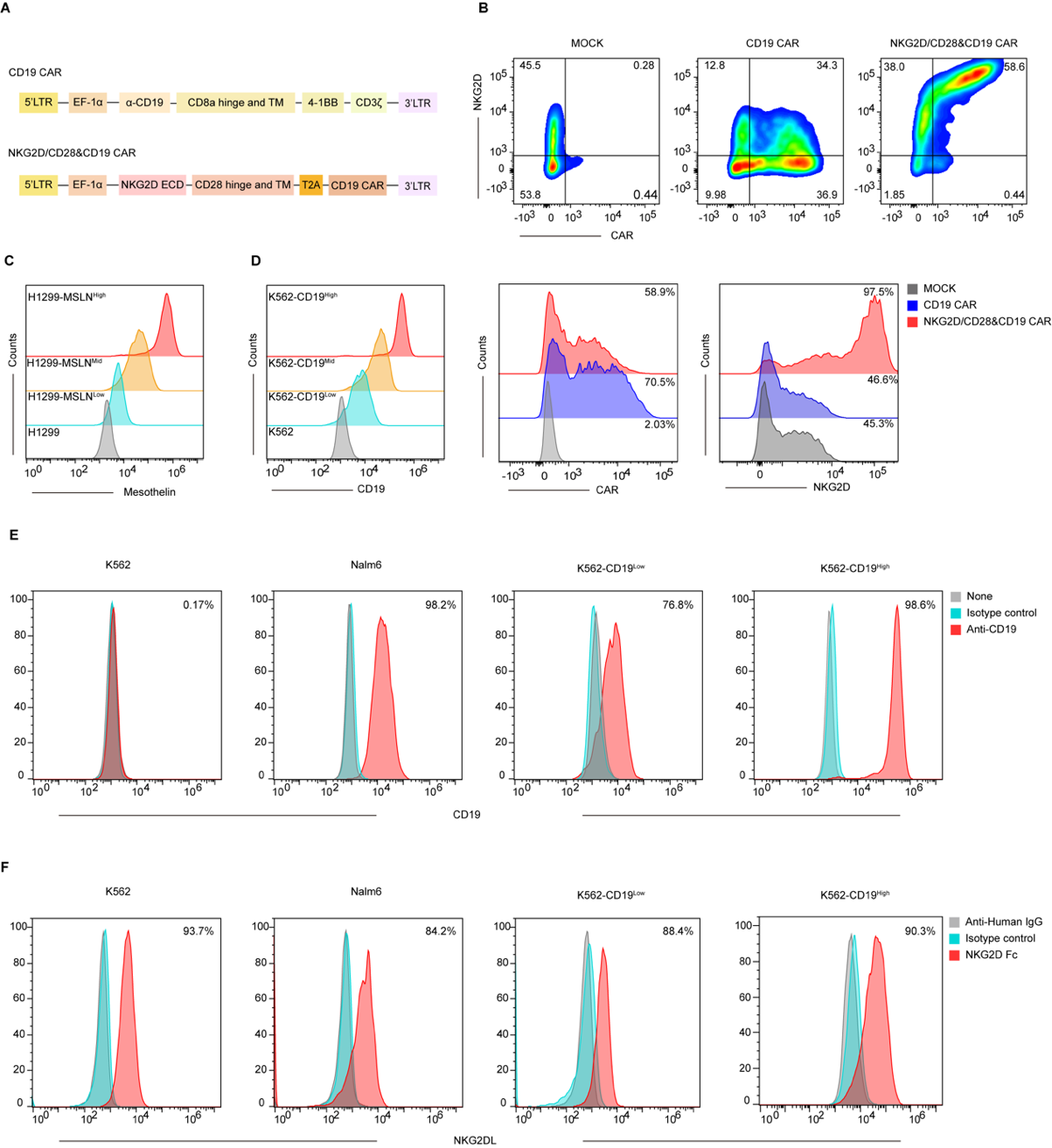
**

**Supplementary Figure.1 Generation and characterization of NKG2D/CD28&CAR-T cells**

**A.** Schematic representation of lentiviral vectors for CD19 CAR and NKG2D/CD28&CD19 CAR. **B.** Representative flow cytometry plots showing the expression of CAR and NKG2D on transduced T cells. **C.** Flow cytometry histograms demonstrating the overexpression of MSLN in H1299-derived cell lines. **D.** Histograms illustrating the overexpression of CD19 in K562-derived cell lines. **E.** Flow cytometry analysis of CD19 and NKG2DL expression on tumor cells, with CD19 detection using anti-CD19 antibody, isotype control (blue histogram), or no antibody control (gray histogram). **F.** Detection of NKG2DLs using recombinant human NKG2D-Fc protein (pink histogram), isotype control (blue histogram), or secondary antibody only (gray histogram).


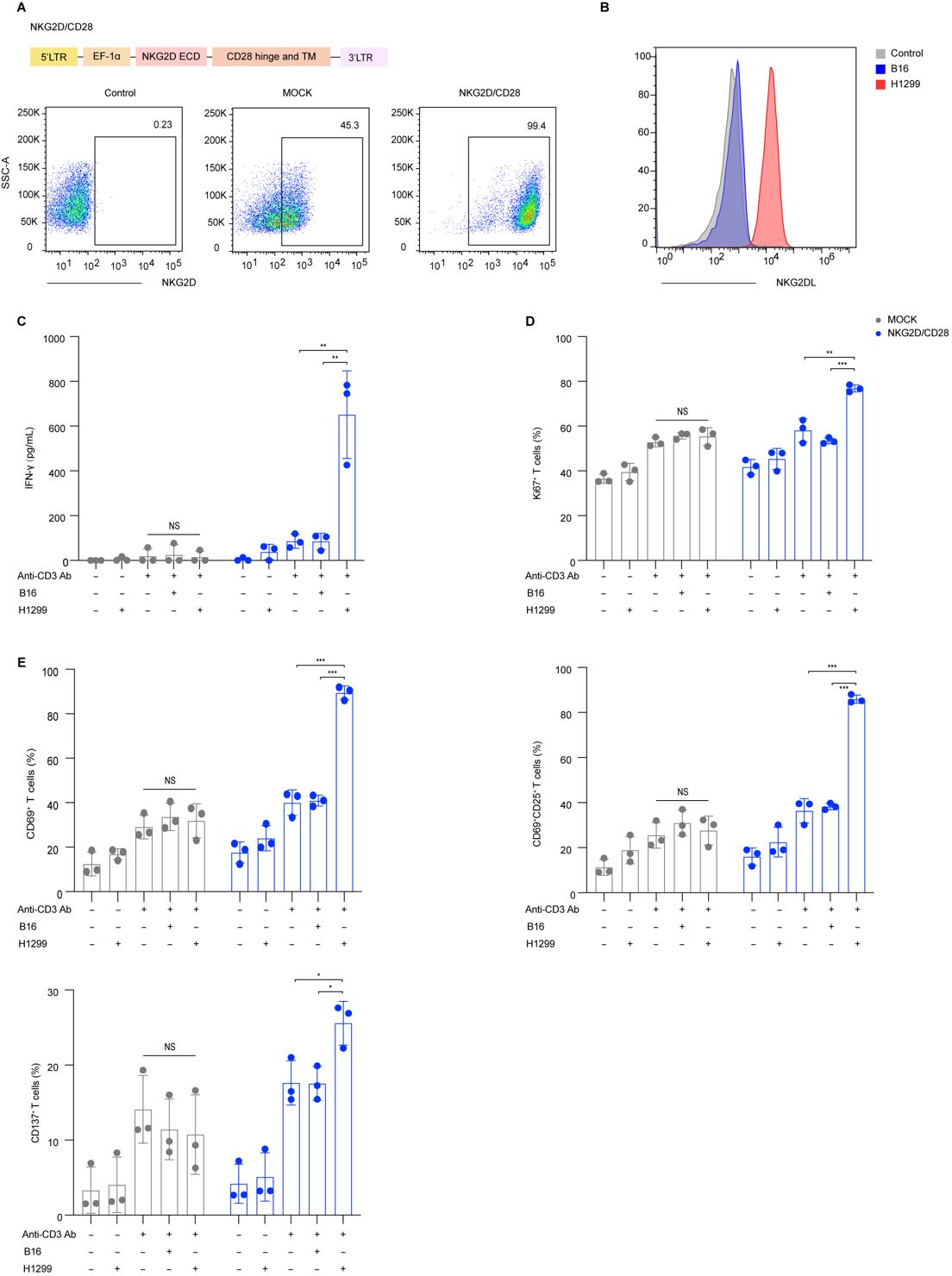


**Supplementary Figure.2 Functional analysis of NKG2D/CD28 CCR-transduced T cells**

**A.** Flow cytometry analysis showing the expression of NKG2D on T cells transduced with the NKG2D/CD28 CCR. **B.** Flow cytometry analysis confirming the expression of NKG2D ligands on H1299 and B16 cell lines. **C.** IFN-γ secretion by NKG2D/CD28 T cells under different stimulation conditions, measured by ELISA. **D.** Ki67 expression in NKG2D/CD28 T cells under different stimulation conditions was detected by flow cytometry. **E.** Activation markers (CD69, CD25, and CD137) expression on NKG2D/CD28 T cells under different stimulation conditions was detected by flow cytometry. Statistical signiﬁcance is deﬁned as follows: *P < 0.05, **P < 0.01, ***P < 0.001.


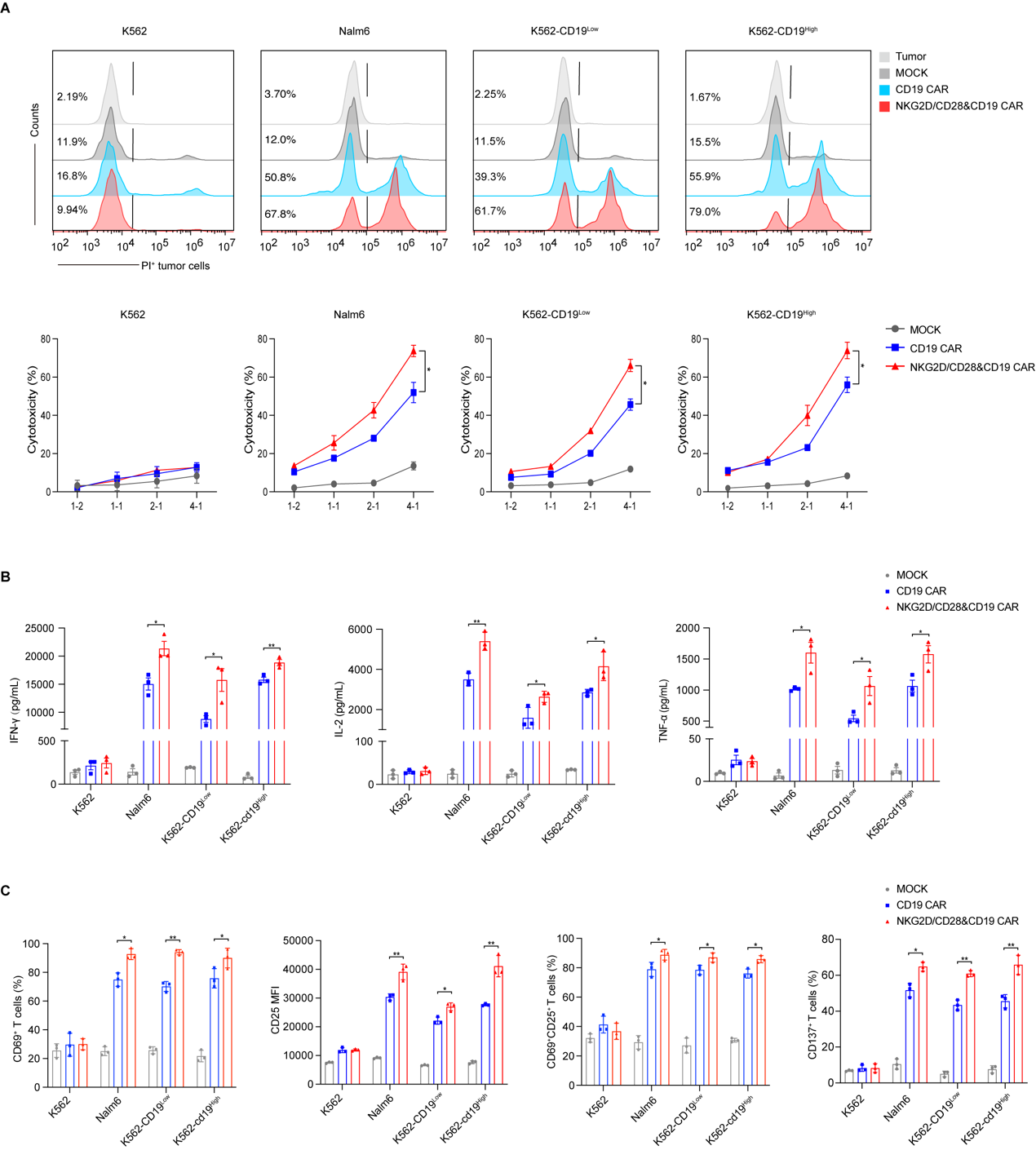


**Supplementary Figure.3 Assessment of cytotoxicity and activation of CAR-T cells against tumor cells**

**A.** Cytotoxicity of CAR-T cells against four types of tumor cells, with representative flow cytometry histograms and corresponding statistical graphs shown. **B.** Levels of cytokines IFN-γ, IL-2 and TNF-α secreted by T cells after co-culture with tumor cells, as detected by ELISA. **C.** Expression of T cell activation markers CD69, CD25, and CD137 after co-culture with tumor cells. Statistical signiﬁcance is deﬁned as follows: *P < 0.05, **P < 0.01, ***P < 0.001.


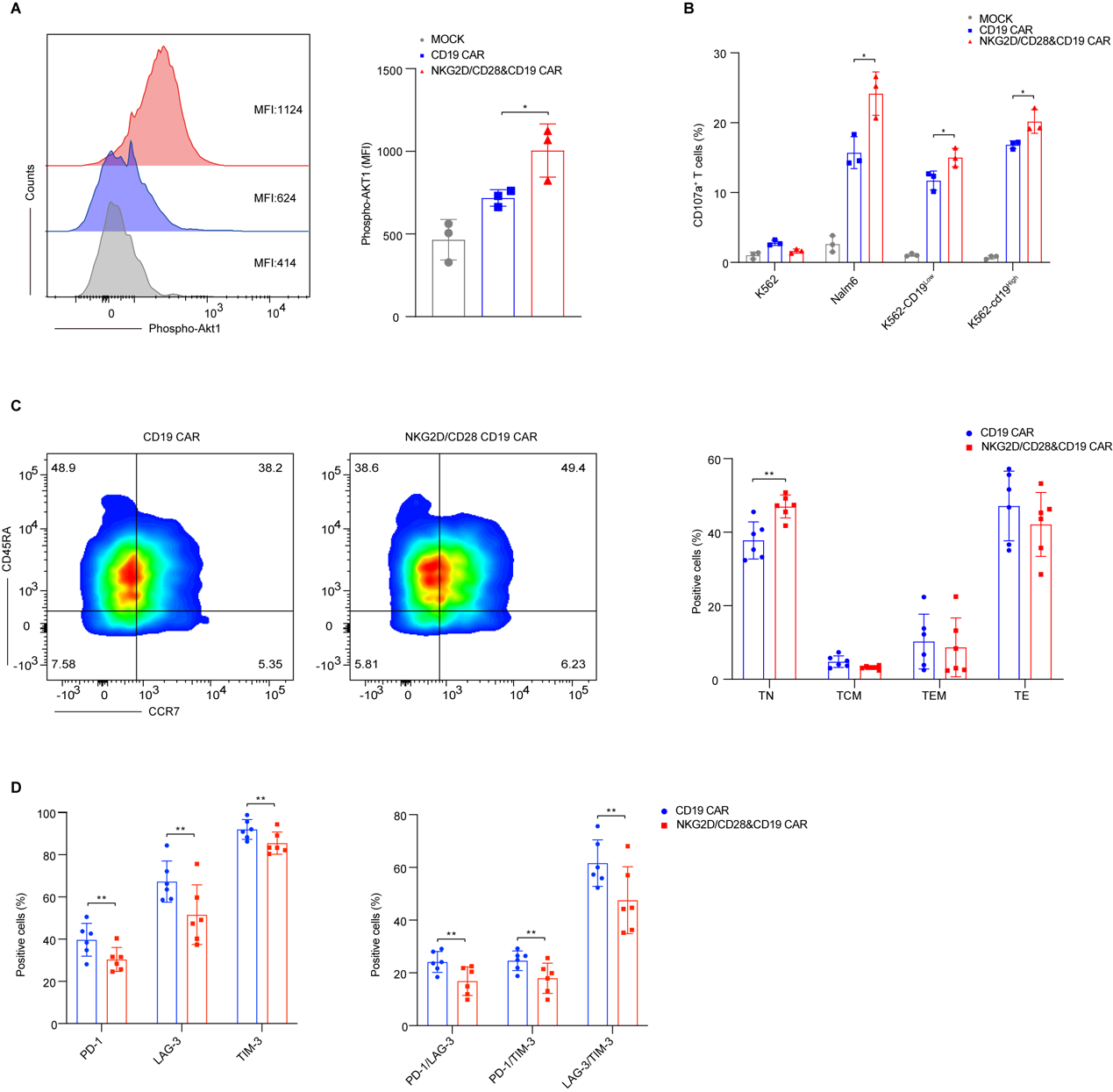


**Supplementary Figure.4 Analysis of CAR-T cell activation and phenotypes following co-culture with tumor cells**

**A.** Flow cytometry detection of phosphorylated Akt1 in CD19 CAR and NKG2D/CD28&CD19 CAR-T cells activated by tumor cells, with representative histograms and statistical graphs of phosphorylated Akt1 MFI shown. **B.** Flow cytometry detection of CD107a-expressing CAR-T cells after co-culture with tumor cells for 4 hours. **C.** Flow cytometry detection of CAR-T cell differentiation phenotypes after co-culture with tumor cells for 24 hours, with representative flow plots on the left and statistical graphs on the right. **D.** Flow cytometry detection of exhaustion markers PD-1, LAG-3 and TIM-3 on CAR-T cells after co-culture with tumor cells for 72 hours. Statistical signiﬁcance is deﬁned as follows: *P < 0.05, **P < 0.01, ***P < 0.001.


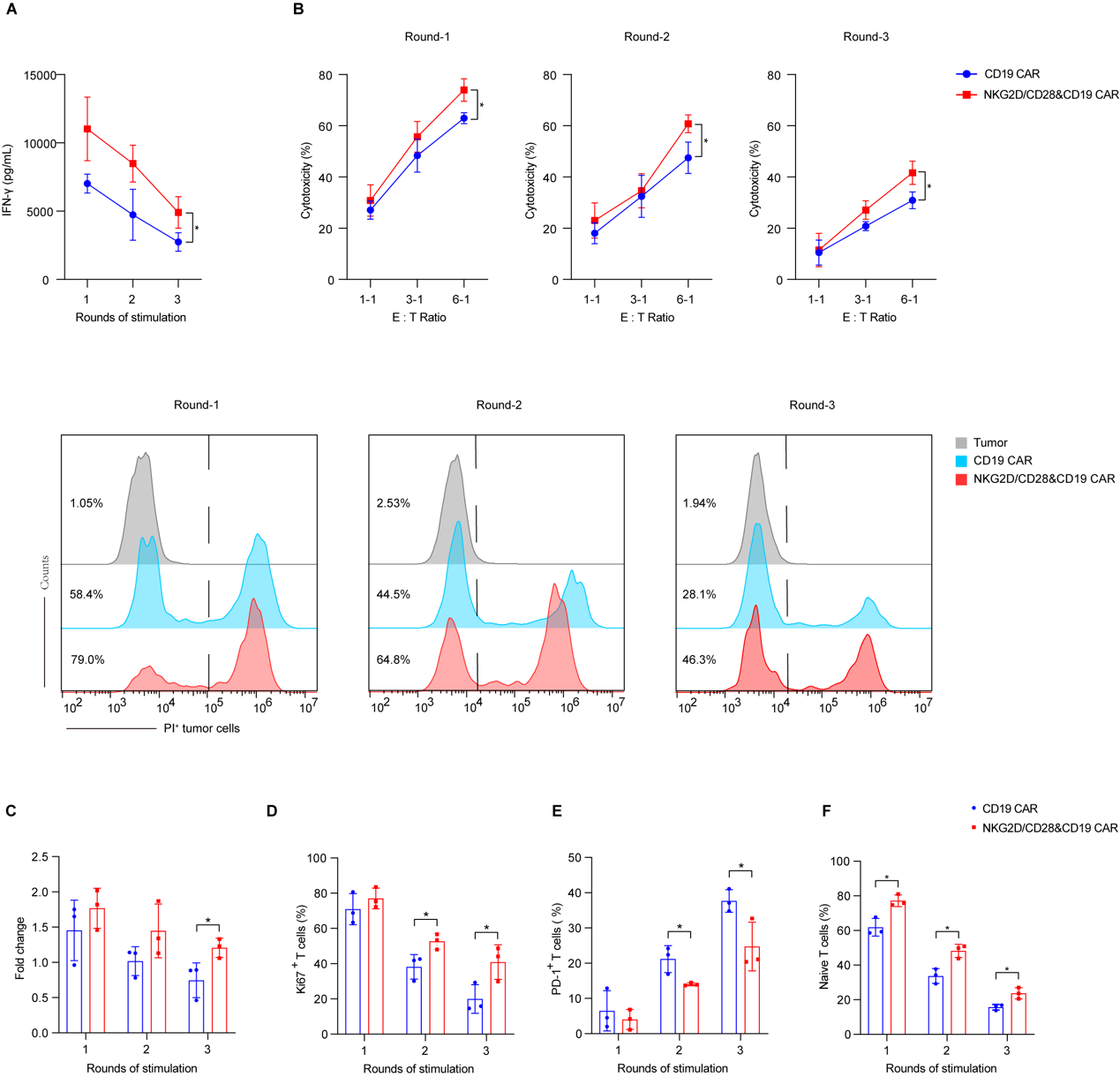


**Supplementary Figure.5 Chronic stimulation of CAR-T cells with mitomycin C-treated tumor tells and subsequent analysis**

**A.** ELISA measurement of IFN-γ secretion levels from CD19 CAR and NKG2D/CD28&CD19 CAR-T cells after repeated stimulation with tumor cells (n=3). **B.** Cytotoxicity of CAR-T cells against tumor cells at various E:T ratios following each round of tumor antigen stimulation, with representative flow cytometry histograms and statistical graphs shown (n=3). **C.** Proliferation of CAR-T cells after each round of antigen stimulation, as indicated by live cell counts. **D.** Flow cytometry detection of Ki67 expression in CAR-T cells following antigen stimulation. **E.** Assessment of PD-1 expression in CAR-T cells by flow cytometry following antigen stimulation. **F.** Flow cytometric quantification of naïve T cell fraction within CAR-T cell populations following antigen exposure. Statistical signiﬁcance is deﬁned as follows: *P < 0.05, **P < 0.01, ***P < 0.001.
